# The PI3K/Akt signaling pathway exerts effects on the implantation of mouse embryos by regulating the expression of RhoA

**DOI:** 10.3892/ijmm.2014.1701

**Published:** 2014-03-17

**Authors:** LIYUAN LIU, YINGXIONG WANG, QIUBO YU

**Affiliations:** 1 College of Basic Medicine, Chongqing Medical University, Yuzhong, Chongqing 400016, P.R. China; 2 Molecular Medical Laboratory, Chongqing Medical University, Yuzhong, Chongqing 400016, P.R. China

**Keywords:** embryo implantation, phosphoinositide 3-kinase/Akt, RhoA, implantation site, inter-implantation site

## Abstract

The aim of this study was to investigate whether the phosphoinositide 3-kinase (PI3K)/Akt signaling pathway affects the implantation of mouse embryos by regulating the expression of RhoA. The expression of PI3K, Akt, phosphorylated (p-)Akt, phosphatase and tensin homolog (PTEN) and RhoA in the uterus of mice on day 5 of pregnancy (D5) and in pseudopregnant mice was examined by quantitative reverse transcription polymerase chain reaction (qRT-PCR), immunohistochemistry and western blot analysis. A functional analysis of these genes was also performed by the intrauterine injection with the PI3K inhibitor, LY294002, on day 2 of pregnancy (D2). The expression levels of PI3K, p-Akt, RhoA at the implantation site were higher than those at the inter-implantation site in the endometrium; however, opposite effects were observed for PTEN expression. The expression levels of the above genes in the pseudopregnant group and in the group injected with the PI3K/Akt inhibitor, LY294002, were markedly lower than those in the pregnant group. Functional experiments revealed that the number of implantation sites had been significantly decreased (P<0.05) following the intrauterine injection of the PI3K inhibitor, LY294002, on day 2 of gestation compared with the contralateral injection of phosphate-buffered saline (PBS). These results suggest that the PI3K/Akt signaling pathway affects embryo implantation by regulating the expression of RhoA.

## Introduction

Embryo implantation is an important step for a successful pregnancy (1). The physiological process involves blastocyst migration, positioning, adhesion, implantation and a series of complex events (2). The interaction between the blastocyst and the receptive endometrium is essential during implantation. This complex and delicate process is regulated by various factors, including the mutual recognition between the trophocyte and the endometrium, the invasion of the trophocyte, early proliferation and differentiation of the embryo and the signal transduction throughout the entire process (3).

RhoA, RhoB and RhoC, Rho subfamily members, present an abnormal expression in a variety of malignant tumors and are thus involved in tumorigenesis (4), affecting the polarities and morphologies of the cells by regulating the aggregation of the actin filament skeleton. They play a role in the migration, invasion and proliferation of tumor cells by affectting cell movement and adhesion in the cell-cell or cell-matrix, and the reconstruction of the extracellular matrix (5,6). Rho subfamily members play an important role in the migration, invasion and proliferation of tumor cells, while the behavior of the embryo implanted in the endometrium is similar with the behavior of tumor cells (7). Thus, RhoA may play an important role in embryo implantation; however, the exact signaling pathway which regulates RhoA remains to be identified.

The phosphatidylinositoi 3-kinase (PI3K)/Akt signaling pathway regulates and controls a variety of cellular functions, such as proliferation, growth and survival (8) Under normal circumstances, the activated PI3K products, phosphatidylinositol (3,4)-bisphosphate [PI(3,4)P2] and phosphatidylinositol 3,4,5-trisphosphate [PI(3,4,5)P3], activated the downstream signaling protein, Akt, as a second messenger. Activated Akt regulates a variety of cellular functions by phosphorylation (9,10). In this process, the activity of the PI3K/Akt signaling pathway is negatively regulated by the lipid-phosphatase, phosphatase and tensin homolog (PTEN), which removes the phosphoric acid from the 3′ and 5′ of PIP3 and converts it into PI(4,5)P2 and PI(3,4)P2 and degrades it (11,12). The aim of this study was to investigate whether the PI3K/Akt signaling pathway plays an important role during embyro implantation, and whether the PI3K/Akt signaling pathway affects embyro implantation by regulating the expression of RhoA. We wished to determine whether the PI3K/Akt signaling pathway mediates signal transduction at the implantation site and the inter-implantation site of the pregnant mice by affecting the expression of RhoA.

## Materials and methods

### Materials

Experimental SPF level Kunming mice (aged 8–10 weeks, weighing 25–30 g) were purchased from the Experimental Animal Center, Chongqing Medical University (Chongqing, China). All animal experiments were approved by the Ethics Committee of Chongqing Medical University. The mice were bred under controlled environmental conditions (light, 14 h; dark, 10 h; temperature range, 22–25°C). The female and male mice were mated at the ratio of 2:1, and a vaginal plug was used to mark the first day of pregnancy (D1). Normal adult male mice were fed for 14 days following a vasectomy (model of pseudopregnancy) and were mated with the female mice at a ratio of 1:2; a vaginal plug was used to mark the first day of pseudopregnancy (PD1). The mice were randomly divided into 3 groups: the first group was the pregnant group, the second group was the pseudopregnant group, and the third group was the experimental group administered the intrauterine injection of the PI3K inhibitor, LY294002. There were 20 mice in each group. The mice were sacrificed by decapitation and the uterus was then removed following the injection of 0.4% trypan blue (0.15 ml) between 8:00–9:00 a.m. on day 5. In each group, 10 mice were used for quantitative reverse transcription polymerase chain reaction (qRT-PCR) and western blot analysis and 10 mice for immunohistochemistry with 4% paraformaldehyde.

### qRT-PCR

A total of 50–100 mg of endometrial tissues (from implantation and inter-implantation sites) obtained (on day 5) from normal pregnant mice, pseudopregnant mice and mice injected with the PI3K/Akt inhibitor was placed in a homogenizer. Total RNA was extracted in accordance with the instructions provided with the TRlzol reagent (Invitrogen Corp., Carlsbad, CA, USA). The D260/D280 ratio was measured using a spectrophotometer to determine the purity and concentration of the RNA. RNA was reverse transcribed into cDNA under the following reaction conditions: 37°C for 15 min and 85°C for 5 sec. Quantitative fluorescence PCR was used to amplify the PI3K, Akt, PTEN and RhoA gene products in a 25 μl reaction system, with the use of SYBR-Green mix (12.5 μl), 1 μl of upstream and downstream primers, cDNA (2 μl) and RNase-free H_2_O (8.5 μl). The reaction conditions were as follows: 95°C for 3 min, 95°C for 10 sec, 60°C for 30 sec, 40 cycles; the detection of the dissolution curve was carried out at 65–95°C. The primer sequences used are presented in Table I. The experiment was repeated 3 times. β-actin was used as the reference gene to determine a normalized arbitrary value for each gene. Relative expression was calculated according to the equation 2^−ΔΔCt^ and statistically analyzed using a t-test.

### Immunohistochemistry

Immunohistochemical measurements of the expression of PI3K, Akt, PTEN and RhoA in the endometrial tissue of the mice in the pregnant group, the pseudopregnant group and the group injected with the PI3K inhibitor were carried out on day 5. The uterus was fixed with 4% paraformaldehyde, dehyderated with graded ethanol, wrapped with xylene transparent paraffin and sliced into sections (5-μm-thick) prior to immunohistochemical staining. Immunohistochemistry was performed using the SP-9001 Reagent kit (Zhongshan Goldenbridge Biotechnology Co., Ltd., Beijing, China). The sections were then stained with primary antibodies to PI3K (1:50; PI3-kinase p110 α antibody, BSA1236; Bioworld Technology, Inc.), Akt [1:80; Akt (P125) antibody, BS3987; Bioworld Technology, Inc.], phosphorylated (p-)Akt [1:80; p-Akt (S473) pAb antibody, BS4006; Bioworld Technology, Inc.], PTEN [1:80; PTEN (D386) antibody, BS5534; Bioworld Technology, Inc.], RhoA [1:50; RhoA (R182) antibody, BS1782; Bioworld Technology, Inc.]. The sections were then examined under a microscope (Olympus BX51) and images were acquired. The intensity of positive expression was determined using Image Pro^®^Plus v. 6.0 software. Phosphate-buffered saline (PBS) was used instead of the primary antibody for the negative control.

### Western blot analysis

Pregnant mice [day 5 of pregnancy (D5)] were injected with 0.4% trypan blue (0.15 ml) into the vena caudalis, and then sacrificed following anesthesia for 10 min. The uterus were isolated to obtain the implantation site and the inter-implantation site in the endometrium. Approximately 100 mg endometrial tissue was obtained from the mice at the implantation site and inter-implantation site; 200 μl protein lysate (Beyotime Institute of Biotechnology, Shanghai, China) and 2 μl of the protease inhibitor, PMSF (100 mmol/l), were then added to the sections after being milled by liquid nitrogen; the sections were then ice bath shock cracked for 20 min at 4°C, and oscillated every 5 min. The tissues were centrifuged at 12,000 rpm for 15 min, and the BCA kit was used to measure the protein content. Protein sample extracts (each approximately 50 μg) were dissolved in 5× loading buffer at a ratio of 4:1, mixed and boiled in water for 10 min; they were then subjected to 10% SDS-PAGE gel electrophoresis, at a constant voltage of 80 V for 2 h; the separated protein was electrically transferred onto PVDF membranes (0.45 μm), and the membranes were incubated for 1 h at room temperature with 3% BSA, and then incubated overnight with primary antibody (the antibody and the proportion of BSA was 1:1,000); the membranes were then washed 3 times with PBST, each time for 5 min. The bands were incubated for 1 h with 3% BSA diluted second antibody at room temperature and washed 3 times with PBST. Finally, the chemiluminescence ECL method was used to develop the observed strip with β-actin as the internal control. Densitometric analysis of gene expression was performed using Quantity One software version 4.6.7.

### Intrauterine injection of the PI3K inhibitor, LY294002

Pregnant mice from the third group were narcotized with 3% sodium pentobarbital on day 2 of pregnancy (D2), and were then administered an intrauterine injection of LY294002, as previously described (13) (concentration, 40 μM; volume, 10 μl), or a contralateral intrauterine injection of isometrical PBS. The mice were then injected with 0.4% trypan blue (0.15 ml) into the vena caudalis on day 5 of pregnancy (D5), and were then sacrificed following anesthesia for 10 min after the injection.

### Statistical analysis

All the experimental data were analyzed using SPSS19.0 statistical software, and a t-test. A value of P<0.05 was considered to indicate a statistically significant difference.

## Results

### qRT-PCR analysis of mRNA expression of the PI3K, Akt, PTEN and RhoA genes at the implantation and inter-implantation sites in the endometrium of pregnant mice on day 5

**Figure 1:**
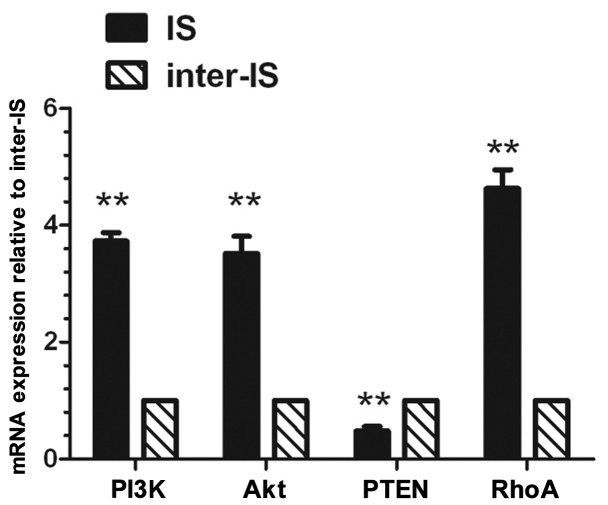
Real-time polymerase chain reaction (PCR) of the mRNA expression levels of PI3K, Akt, PTEN and RhoA at the implantation site and the inter-implantation site in the endometrium. β-actin was used as an internal control. The experiment was performed 3 times. Three mice were used in each experiment. The analysis was performed on day 5. ^**^P<0.01 indicates statistical significance between the implantation site and inter-implantation site in the endometrium. IS, implantation site; inter-IS, inter-implantation site.

qRT-PCR (Fig. 1) revealed that the mRNA expression levels of PI3K, Akt and RhoA at the implantation site in the endometrium on day 5 of pregnancy were significantly higher than those at the inter-implantation site (P<0.01). However, the mRNA expression level of PTEN at the implantation site was significantly lower than that at the inter-implantation site (P<0.01).

### Western blot analysis of PI3K, Akt, p-Akt, PTEN and RhoA at the implantation and inter-implantation sites in the endometrium of pregnant mice on day 5

**Figure 2:**
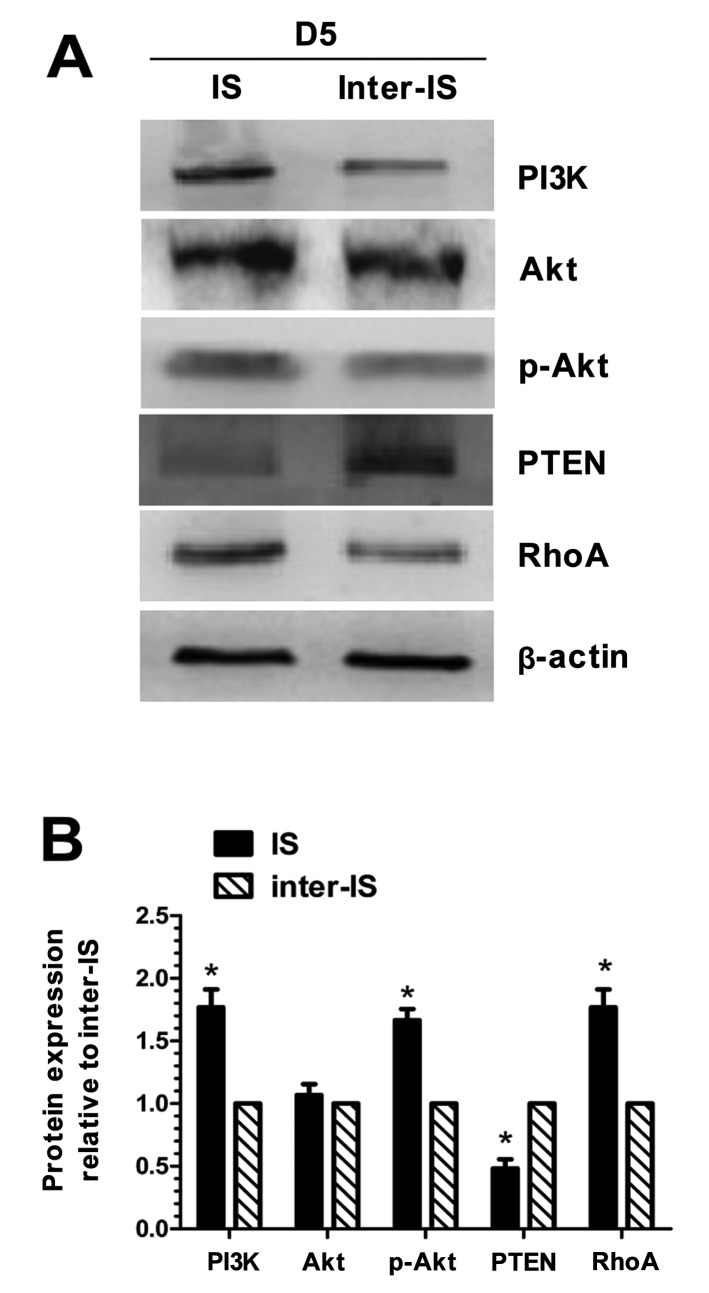
Western blot analysis of the protein expression levels of PI3K, Akt, p-Akt, PTEN and RhoA at the implantation site and inter-implantation site in the endometrium. (A) Western blot analysis electrophoresis graph. (B) Relative densitometric analysis of these proteins between the implantation site and the inter-implantation site. A single representative experiment is shown. The experiment was performed 3 times. Three mice were used in each experiment. The analysis was performed on day 5. ^*^P<0.05 indicates statistical significance between the implantation site and inter-implantation site in the endometrium. IS, implantation site; inter-IS, inter-implantation site.

Western blot analysis (Fig. 2) revealed that the protein expression of PI3K, p-Akt and RhoA at the implantation site in the endometrium of the mice was significantly higher than that at the inter-implantation site (P<0.05). As regards the expression of Akt, no significant difference was observed between the implantation site and the inter-implantation site in the endometrium. The expresion of PTEN at the implantation site in the endometrium was lower than that at the inter-implantation site (P<0.05).

### Location of PI3K, Akt, p-Akt, PTEN and RhoA expression at the implantation and inter-implantation sites in the endometrium of pregnant mice on day 5

**Figure 3:**
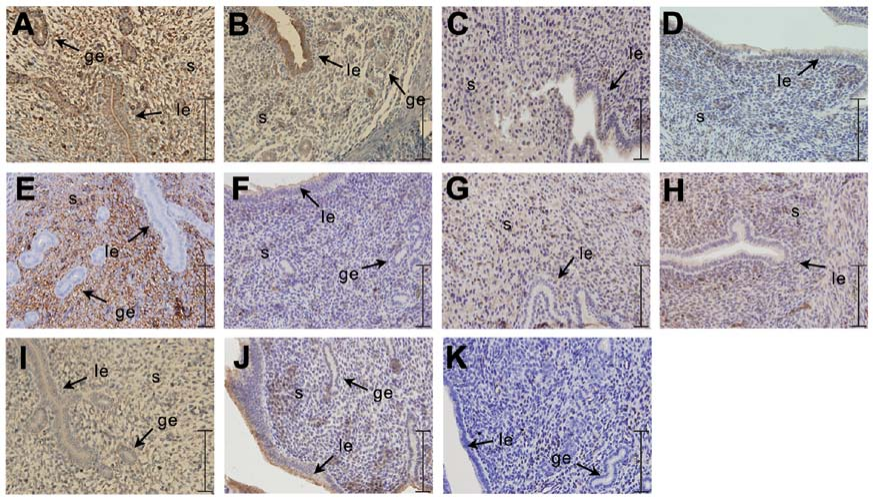
Immunohistochemical analysis of the expression of PI3K/Akt signaling pathway-related genes and RhoA at the implantation site and the inter-implantation site on day 5 of pregnant (D5) mice. The experiment was performed 3 times. Three mice were used in each experiment. (A, C, E, G and I) Representative images of implantation site; (B, D, F, H and J) representative images of inter-implantation site; (K) negative control. (A and B) PI3K, (C and D) Akt, (E and F) p-Akt, (G and H) PTEN, (I and J) RhoA. Yellow-brown color represents positive staining. IS, implantation site; inter-IS, inter-implantation site; le, luminal epithelium; ge, glandular epithelium; s, stromal cells; bar, 125 μm.

Immunohistochemistry (Fig. 3) revealed that the gene expression of PI3K, Akt, p-Akt and RhoA at the implantation site in the uterus of pregnant mice on day 5 was significantly higher than that at the inter-implantation site; however, the opposite effect was observed for PTEN. In the endometrium of pregnant mice on day 5, PI3K expression was strongly positive in the glandular epithelium and stromal cells at the implantation site, while at the inter-implantation site, PI3K protein was significantly expressed in the glandular epithelium and weakly expressed in the stromal cells. Akt protein was significantly expressed in the stromal cells and luminal epithelium of the uterine implantation site, while it was weakly expressed in the luminal epithelium of the inter-implantation site. p-Akt protein was strongly expressed in the stromal cells of the uterine implantation site, and weakly expressed in the luminal epithelium of the uterine inter-implantation site. PTEN protein was expressed in the stromal cells of the implantation site in the endometrium of the pregnant mice on day 5, and weakly expressed in the stromal cells and luminal epithelium of the mouse endometrium inter-implantation site. RhoA was highly expressed in the stromal cells and glandular epithelium at the implantation site in the endometrium of mice, and significantly expressed in the luminal epithelium at the inter-implantation.

### qRT-PCR of PI3K/Akt and RhoA expression in endometrial tissue from pseudopregnant mice on day 5

**Figure 4:**
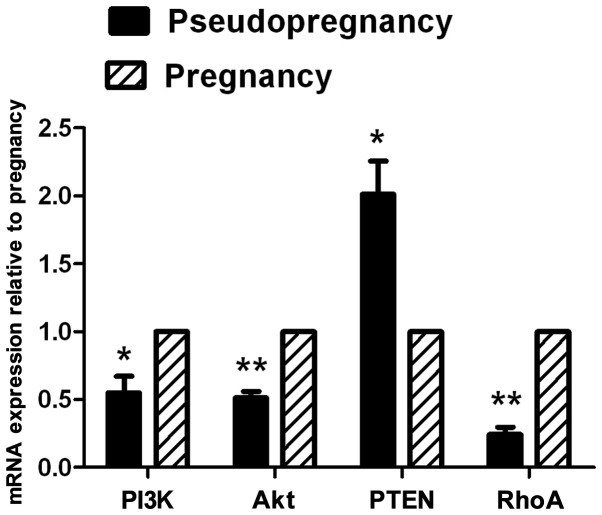
qRT-PCR of the mRNA expression levels of PI3K, Akt, PTEN and RhoA in the endometrium in the pseudopregnant group and the pregnant group. β-actin was used as an internal control. The experiment was performed 3 times. Three mice were used in each experiment. ^*^P<0.05 and ^**^P<0.01 indicate statistical significance between the pseudopregnant group and the pregnant group.

The mRNA expression of PI3K, Akt and RhoA in the endometrial tissue from pseudopregnant mice (Fig. 4) was lower than that in the pregnant group (P<0.01); the expression of the PTEN gene in the endometrial tissue from pseudopregnant mice was higher than that in the pregnant group (P<0.05).

### Immunohistochemistry of PI3K, Akt, p-Akt, PTEN and RhoA expression in endometrial tissue from pseudopregnant mice

**Figure 5:**
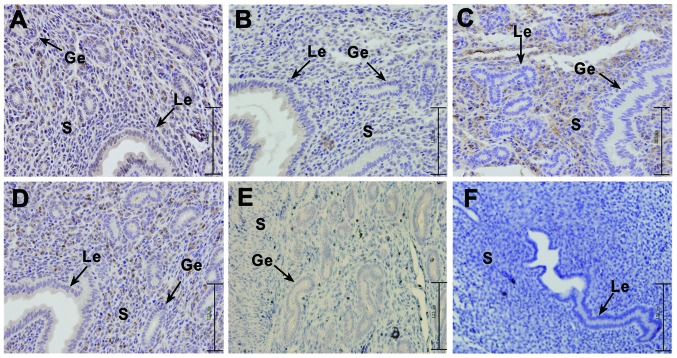
Immunohistochemical analysis of the expression of PI3K/Akt singnaling pathway-related genes (PI3K, Akt, p-Akt and PTEN) and RhoA on day 5 in endometrial tissue from pseudopregnant mice. The experiment was performed 3 times. Three mice were used in each experiment. (A) Representative image of PI3K, (B) representative image of Akt, (C) representative image of p-Akt, (D) representative image of PTEN, (E) representative image of RhoA, (F) representative image of negetive control. Yellow-brown color represents positive staining. le, luminal epithelium; ge, glandular epithelium; s, stromal cells; bar, 125 μm.

In the pseudopregnant mice on day 5, (PD5) PI3K was weakly expressed in the luminal epithelium, glandular epithelium and stromal cells, and Akt was weakly expressed in the luminal epithelium, glandular epithelium and stromal cells. p-Akt was weakly expressed in stromal cells and PTEN was weakly expressed in the stromal cells and luminal epithelium, as well as in the glandular epithelium. RhoA was not expressed in the endometrium of the pseudopregnant mice on day 5 (Fig. 5).

### qRT-PCR of PI3K, Akt, p-Akt, PTEN and RhoA expression at the implantation site in endometrial tissue following the intrauterine injection of PI3K inhibitor

**Figure 6:**
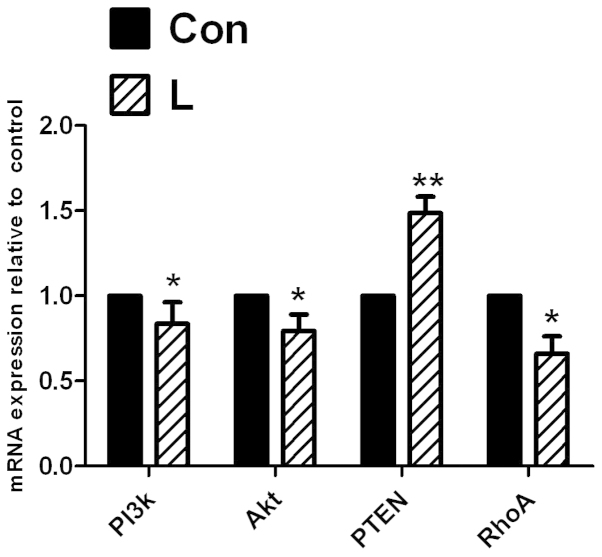
qRT-PCR of the mRNA expression of PI3K, Akt, PTEN and RhoA at the implantation site in the inhibitor group injected with LY294002 (L) and the control group (Con; not injected). β-actin was used as the internal control. The experiment was performed 3 times. Three mice were used in each experiment. ^*^P<0.05 and ^**^P<0.01 indicates statistical significance between the pregnant group and the inhibitor group.

The expression of PI3K and Akt was significantly decreased (P<0.05) when comparing the inhibitor group with the control group (Fig. 6) following the intrauterine injection of the PI3K inhibitor, LY294002, and PTEN expression was significantly increased (P<0.01); RhoA expression was significantly decreased (P<0.05).

### Western blot analysis of PI3K, Akt, p-Akt, PTEN and RhoA expression at the implantation site in endometrial tissue following the intrauterine injection of PI3K inhibitor

**Figure 7:**
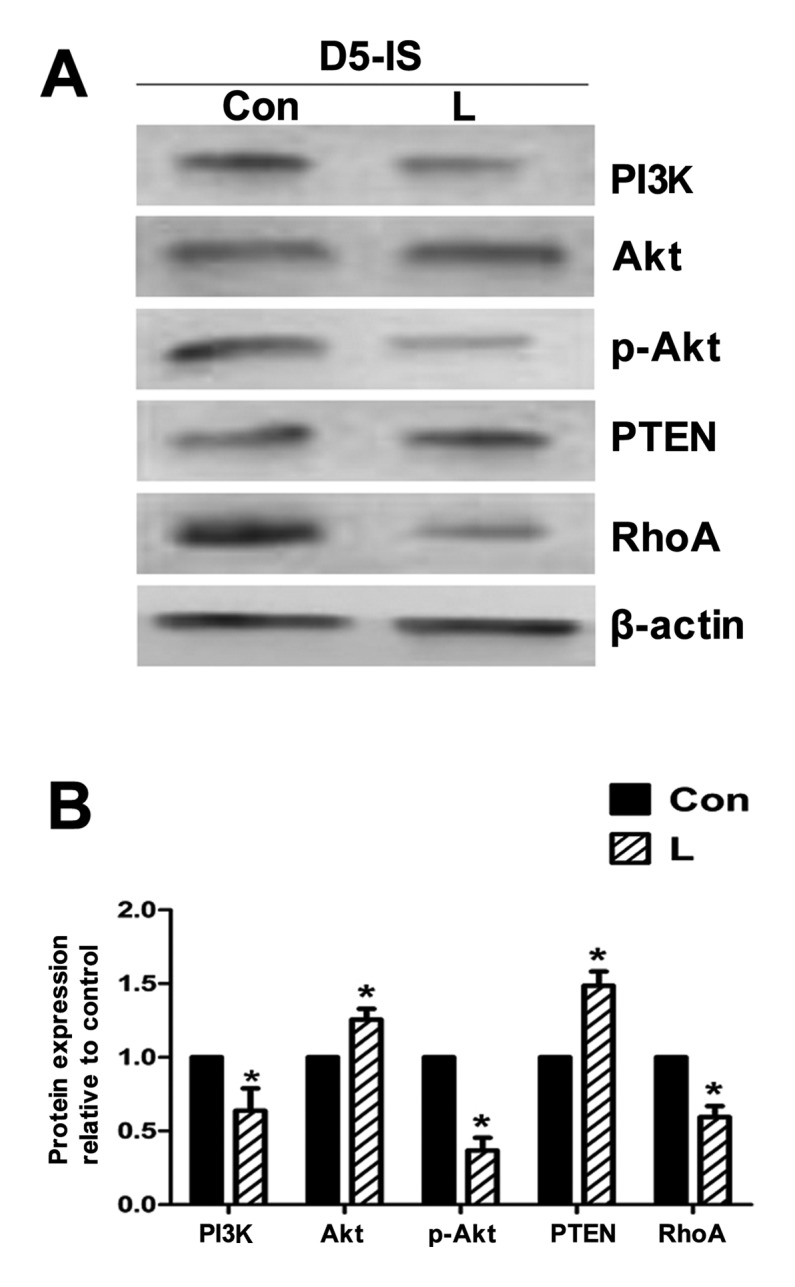
Western blot analysis of the expression of PI3K, Akt, p-Akt, PTEN and RhoA in the inhibitor group injected with LY294002 (L) and the control group (Con; not injected). β-actin was used as the internal control. (A) Western blot analysis electrophoresis graph. (B) Relative densitometric analysis of these proteins between the inhibitor group and the control group. The experiment was performed 3 times. Three mice were used in each experiment. ^*^P<0.05 indicates statistical significance between the inhibitor group and the control group at pregnancy day 5.

The expression of Akt showed no significant alteration; the expression of PI3K and p-Akt was decreased in the group injected with the PI3K inhibitor, LY294002, compared with the control group. The expression of PTEN was significantly increased, and RhoA expression was significantly decreased (Fig. 7) following the intrauterine injection of the PI3K inhibitor, LY294002.

### Immunohistochemistry of PI3K, Akt, p-Akt, PTEN and RhoA expression at the implantation site in endometrial tissue following the intrauterine injection of PI3K inhibitor

**Figure 8:**
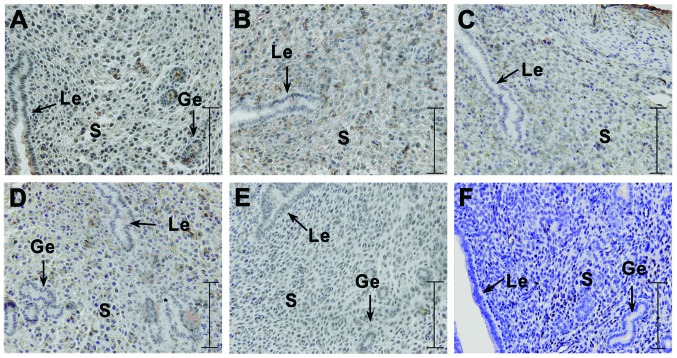
Immunohistochemical analysis of the expression of PI3K, Akt, p-Akt, PTEN and RhoA at the implantation site in the group injected with the PI3K inhibitor in the uterus of pregnancy mice on day 5. Yellow-brown color represents positive staining. (A) Representative image of PI3K; (B) representative image of Akt; (C) representative image of p-Akt; (D) representative image of PTEN; (E) representative image of RhoA. The experiment was performed 3 times. Three mice were used in each experiment. Le, luminal epithelium; ge, glandular epithelium; s, stromal cells; bar, 125 μm.

The expression of PI3K, Akt and p-Akt was significantly decreased in the group injected with the inhibitor compared with that in the non-injected control group. PTEN expression in the inhibitor group was significantly increased (Fig. 8) following the intrauterine injection of the PI3K inhibitor, LY294002. The expression of RhoA was decreased. In addition, the locations of the expression of the above genes had not changed, and the genes were mainly expressed in the stromal cells.

### Effect of PI3K/Akt inhibitor on embryo implantation

**Figure 9:**
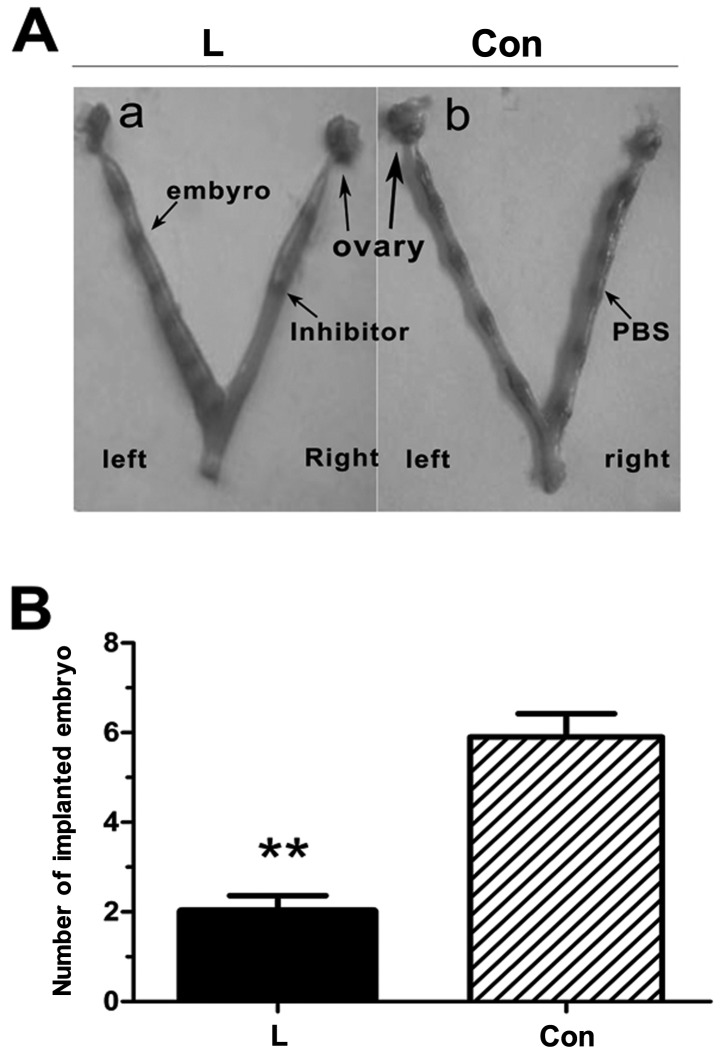
(A) The uterine of the mice in 2 independent experiments. (a) Right-side injection of the inhibitor, LY294002, into the uterus of the mice. (b) Right-side injection of phosphate-buffered saline (PBS) into the uterus of the mice as a control; the left mouse embryonic implantation was significantly lower than that of the right. (B) Measurement of the number of mouse embryo implantations between the treatment group and the control group, and the number of implantations in the treatment group (L; injected with LY294002) was significantly lower than that of the control group (Con); ^**^P <0.01.

The unilateral intrauterine injection of PBS had no effect on embryo implantation, whereas embryo implantation was reduced following the contralateral intrauterine injection of the PI3K inhibitor, LY294002 (Fig. 9), and the results were statistically significant (P<0.01).

## Discussion

Embryo implantation is an important event which relates to the continuation of species. Successful embryo implantation requires effective maternal-fetal dialogue, namely, mutual recognition and adhesion between the blastocyst with the ability to implant and the receptive endometrium (1). Uterine receptivity is considered as the ‘implantation window’, and the uterine microenvironment at this time helps to support blastocyst growth, adhesion response and embryo implantation (14). For pregnant mice, the 4th to 5th day of pregnancy is considered to be the ‘implantation window’ (15). In the process of embryo implantation, all elements in the uterus, including the luminal epithelium, the glandular epithelium and the stromal cells will go through the process of continuous proliferation and differentiation with the embryo adhering to the luminal epithelium and implanting into the stromal cells (16). The endometrial luminal and glandular epithelial cells of mice undergo a proliferative state on days 1 and 2 of pregnancy (D1 and D2). With pregnancy progressing, they exit from the cell cycle and enter a differentiation program that allows them to transit a receptive state. The stromal cells adjacent to the epithelium then begin to proliferate on day 3 and this proliferation becomes widespread following embryo attachment to the receptive luminal epithelium on day 4 of pregnancy. As the embryos invade through the luminal epithelium into the stromal compartment, the stromal cells differentiate into secretory decidual cells, which support further growth and development of the implanted embryos until placentation ensues (16–19).

The endometrium has different adaptive responses in different periods during the embryo implantation process, and its changes are regulated by various cytokines and growth factors (20). During mammalian preimplantation, the developing embryo is dependent on signals generated by growth factors which are known to regulate cell proliferation and differentiation in an autocrine and paracrine manner by means of the endometrial microenvironment (15,21). A large number of PI3K/Akt signaling pathway activated receptors are present in the embryo during the pre-implantation stage (22). In this study, we found that the activation of the PI3K/Akt signaling pathway during the embryo ‘implantation window’ may be activated by the endometrial signaling pathway caused by the activated embryo adhesion. Additionally, RhoA is involved in the regulation of the endometrium. There are a number of signaling factors and signaling pathways involved in embryo implantation; however, the molecular mechanisms involved remain unclear (23,24). As shown in the study by Vanhaesebroeck *et al* (25), the p110α isoform of PI3K plays an important role during embyro implantation; thus, we selected p110α in our study. We found that PI3K expression was strongly positive in the glandular epithelium and stromal cells at the implantation site in the endometrium on day 5 of pregrancy (D5), while PI3K was significantly expressed in the glandular epithelium and weakly expressed in the stromal cells at the inter-implantation site. Akt was significantly expressed in the stromal cells and luminal epithelium at the implantation site, while it was weakly expressed in the luminal epithelium at the inter-implantation site. The p-Akt protein was strongly expressed in the stromal cells at the implantation site, and weakly expressed in the luminal epithelium at the inter-implantation site. The PTEN protein was expressed in the stromal cells at the implantation site in the endometrium on D5, and weakly expressed in the stromal cells and luminal epithelium of the endometrium at the inter-implantation site. RhoA was highly expressed in the stromal cells and glandular epithelium of the mouse endometrium at the implantation site, and significantly expressed in the luminal epithelium of the mouse uterine inter-implantation site. This expression characteristic may be consistent with its functions.

The PI3K/Akt signaling pathway has frequently appeared in a variety of human cancers, such as non-small cell lung cancer (26), breast cancer (27), prostate cancer (9), ovarian cancer (28) and other physiological processes, such as epithelial-mesenchymal transition and hippocampal cell multiplication in tumor development and cancer (26,29). The PI3K/Akt signaling pathway regulates a variety of critical cellular functions, such as proliferation, growth, survival, apoptosis, tumor growth and angiogenesis (8,9). Riley *et al* (21) found that the activation of the PI3K signaling pathway plays an important role in glucose metabolism of the mouse embryo and in embryonic survival; the PI3K/Akt signaling pathway is also crucial during the pre-implantation stage (22). Our study demonstrates that the PI3K/Akt signaling pathway plays an important role during the ‘implantation window’ in mice.

RhoA belongs to the small molecule G protein superfamily, which is widely expressed in different types of cells and tissues. With a variety of biological functions, RhoA plays an important role in the regulation of the actin cytoskeleton, which is mainly involved in the reorganization of the cytoskeleton, cell migration and adhesion, cell polarization and activation and DNA transcription, as well as other functions (30,31). In the reproductive field, Melendez *et al* found that RhoA was essential in mouse embryonic fibroblastic mitosis (9). In our study, we found that when the PI3K inhibitor, LY294002, was used, the expression of RhoA was reduced. At the same, we also used the inhibitor, wortmannin, in our experiments, and observed a similar trend. The specificity of LY294002 to PI3K is higher than that of wortmannin; thus, we only showed the results obtained for LY294002. When we obtained the above results, we hypothesized that RhoA expression is involved during the ‘implantation window’, and may be regulated by the PI3K/Akt signaling pathway.

During the embryo implantation period, embryos which are capable of implantation by means of the endometrial microenvironment release a variety of cytokines, such as integrin and epidermal growth factor (EGF) in an autocrine or paracrine manner. These cytokines activate the expression of signaling pathway-related genes. In this study, the expression levels of the PI3K/Akt signaling pathway-related genes, PI3K, Akt and p-Akt, at the implantation site in the endometrium were higher than those at the inter-implantation site, and the location of their expression was basically the same, mainly strongly positive in the stromal cells; however, the expression of PTEN showed an opposite trend. The expression levels and the distribution characteristics of the PI3K/Akt signaling pathway-related genes at the implantation and inter-implantation sites in the endometrium suggest that the PI3K/Akt signaling pathway is involved during early embryo implantation, particularly during the ‘implantation window’.

In addition, the expression level of RhoA at the implantation site in the endometrium was higher than that at the inter-implantation, which was basically consistent with the expression levels, expression sites and expression time of the PI3K and Akt genes, suggesting that there may be some type of association between the PI3K/Akt signaling pathway and RhoA. This suggests that the mRNA and protein levels of the signaling pathway-related genes and RhoA may be activated by embryo adhesion. In order to confirm this hypothesis, we designed the pseudopregnant group. We found that the expressions of PI3K, p-Akt and RhoA in the pseudopregnant group was lower than that in the pregnant group, which confirmed our hypothesis. To further confirm the association between the PI3K/Akt signaling pathway and RhoA in the embryo ‘implantation window’, we used the PI3K/Akt signaling pathway inhibitor, LY294002. We found that the expression level of RhoA was significantly decreased, and the number of embryo implantations was reduced following the application of the inhibitor, which to some extent reflected that the PI3K/Akt signaling pathway may regulate RhoA expression in the process of the embryo implantation. This suggests that the PI3K/Akt signaling pathway at the implantation site in the endometrium promotes the migration and decidualization of the stromal cells by regulating the expression of RhoA under normal circumstances, thereby contributing to the implantation of the embryo; when the PI3K/Akt signaling pathway was inhibited, the expression level of RhoA was decreased follwoing the injection of the inhibitor, LY294002, which was not conducive to the migration and decidualization of endometrial stromal cells, thereby not conducive to the implantation of the embryo, thus reducing the number of embryo implantations. The PI3K/Akt signaling pathway itself may affect embryo implantation by affecting cell proliferation (22); RhoA can also regulate the expression of PI3K/Akt through the RhoA-ROCK-PTEN signaling pathway in mouse osteoblasts and may affect cell proliferation (13). This suggests that a dual-direction regulation may exist between PI3K/Akt and RhoA in the process of embryo implantation, but this was not further confirmed in this study. Further studies are required to determine the mechanisms through which the PI3K/Akt signaling pathway regulates the expression of RhoA and affects embryo implantation, and whether there are other genes which can regulate embryo implantation.

## Figures and Tables

**Table I:** Primer sequences used for qRT-PCR.

Gene	Primer sequence
PI3K	F: 5′-CTCTCCTGTGCTGGCTACTGT-3′ R: 5′-GCTCTCGGTTGATTCCAAACT-3′
Akt	F: 5′-ATCCCCTCAACAACTTCTCAGT-3′ R: 5′-CTTCCGTCCACTCTTCTCTTTC-3′
PTEN	F: 5′-CATTGCCTGTGTGTGGTGATA-3′ R: 5′-AGGTTTCCTCTGGTCCTGGTA-3′
RhoA	F: 5′-AGCTTGTGGTAAGACATGCTTG-3 R: 5′-GTGTCCCATAAAGCCAACTCTAC -3′
β-actin	F: 5′-CCTGAGGCTCTTTTCCAGCC-3′ R: 5′-TAGAGGTCTTTACGGATGTCA ACGT-3′

F, forward; R, reverse.
